# Attributable risk of lung cancer deaths due to indoor radon exposure

**DOI:** 10.1186/s40557-016-0093-4

**Published:** 2016-02-26

**Authors:** Si-Heon Kim, Won Ju Hwang, Jeong-Sook Cho, Dae Ryong Kang

**Affiliations:** Department of Preventive Medicine and Public Health, Ajou University School of Medicine, Suwon, Korea; College of Nursing Science, East-west Nursing Research Institute, Kyung Hee University, Seoul, Korea; Pharmaceutical Benefits Department, Health Insurance Review & Assessment Service, Seoul, Korea; Department of Humanities and Social Medicine, Ajou University School of Medicine, Suwon, Korea

**Keywords:** Attributable risk, Lung cancer, Mitigation, Radon, Smoking

## Abstract

Exposure to radon gas is the second most common cause of lung cancer after smoking. A large number of studies have reported that exposure to indoor radon, even at low concentrations, is associated with lung cancer in the general population. This paper reviewed studies from several countries to assess the attributable risk (AR) of lung cancer death due to indoor radon exposure and the effect of radon mitigation thereon. Worldwide, 3–20 % of all lung cancer deaths are likely caused by indoor radon exposure. These values tend to be higher in countries reporting high radon concentrations, which can depend on the estimation method. The estimated number of lung cancer deaths due to radon exposure in several countries varied from 150 to 40,477 annually. In general, the percent ARs were higher among never-smokers than among ever-smokers, whereas much more lung cancer deaths attributable to radon occurred among ever-smokers because of the higher rate of lung cancers among smokers. Regardless of smoking status, the proportion of lung cancer deaths induced by radon was slightly higher among females than males. However, after stratifying populations according to smoking status, the percent ARs were similar between genders. If all homes with radon above 100 Bq/m^3^ were effectively remediated, studies in Germany and Canada found that 302 and 1704 lung cancer deaths could be prevented each year, respectively. These estimates, however, are subject to varying degrees of uncertainty related to the weakness of the models used and a number of factors influencing indoor radon concentrations.

## Background

Radon is a chemically inert radioactive gas of natural origin that is produced from uranium and radium in rocks and soils throughout the earth’s crust. Outdoors, radon is of no concern to human health, because it is quickly diluted by atmospheric mixing [1]. However, radon can accumulate to harmful levels in confined spaces such as homes and workplaces. Inhalation of high levels of radon, a source of radioactivity, can induce DNA mutation and increase the risk of cancer by depositing decay products in the lung epithelium [2]. In 1988, the International Agency for Cancer Research declared radon to be carcinogenic for humans and classified as a proven human carcinogen [3].

Radon is the second most common cause of lung cancer after smoking [4]. Long-term exposures to radon have been linked to lung cancer in several epidemiological studies [5]. In the sixteenth century, mortality from respiratory disease increased among certain groups of underground miners in Central Europe, and the primary cause of deaths was first suspected as radon-related lung cancer in the twentieth century [4]. Evidence on health effects of radon comes mainly from epidemiological studies of underground miners exposed to high concentrations of radon, which has consistently been shown to be related to an increased risk of lung cancer for both smokers and non-smokers [2]. Beginning in the 1980s, a large number of studies set out to investigate associations between lung cancer and exposure to indoor radon among the general population. However, these studies failed to provide definitive results, mainly because of the small number of study participants; some showed a significant relationship between indoor radon exposure and lung cancer [6], while others did not [7]. Therefore, pooled-analyses were undertaken to ascertain associations in the general populations of Europe, North America, and China [5, 8–11]. These collaborative analyses presented similar results on a positive association between indoor radon exposure and lung cancer risk.

Indoor radon may constitue a significant and potentially preventable risk factor for lung cancer. Measuring health risks can be useful to public health policy making and the allocation of available resources. The population risk of radon-induced lung cancer is assessed by attributable risk (AR). AR is a measure of how much of the disease risk is attributable to a certain exposure, and thus, indicates the potential for prevention if the exposure could be eliminated [12]. Explicitly, the AR of lung cancer deaths due to indoor radon exposure refers to the proportion of lung cancer deaths that could be prevented if indoor radon concentrations were remediated to outdoor levels [13]. In fact, since most homes are deemed to have low levels of radon, the majority of lung cancer deaths attributable to radon would occur among persons exposed to indoor radon concentrations below commonly used reference levels [4]. Therefore, strategies to mitigate indoor radon levels are necessary for public health, and quantitative analyses would help in determining appropriate reference levels thereof. In this review, we summarized the results of studies from several countries on ARs of lung cancer deaths due to indoor radon exposure and the effects of radon mitigation.

## Review

### Lung cancer deaths attributable to indoor radon exposure per country

Tables 1 and 2 show the estimated percentages and numbers of lung cancer deaths attributable to indoor radon exposure in American, European, and Asian countries [1, 2, 14–21]. The percent AR of lung cancer deaths due to radon exposure is estimated to be lie between 3 % (United Kingdom) and 20 % (Sweden). These calculations suggest that of all lung cancer deaths worldwide, 3–20 % may be caused by indoor radon exposure. The number of lung cancer deaths attributed to radon exposure ranges from 150 (Netherlands) to 40,477 (South Korea). The wide variation of the estimates among countries may be due to the exposure-response relation model used and the overall number of lung cancer deaths in each country. These findings support that indoor radon exposure poses a significant hazard to public health. Indeed, radon-induced lung cancer deaths may be greater than deaths from other cancers. As an example, the estimated number of lung cancer deaths due to radon exposure in the United States is greater than the annual number of deaths for several cancers including malignant neoplasms of the ovaries, liver, brain, stomach, or melanoma [4].

**Table 1:** Percentage of lung cancer deaths attributable to indoor radon according to smoking status and gender

Country (reference)	Mean indoor radon (Bq/m^3^)	Model used in risk estimation	Ever-smokers			Never-smokers			Ever- and never-smokers		
			Male	Female	Total	Male	Female	Total	Male	Female	Total
United States											
([2], 1999)	46	BEIR VI, EAC	12.5	13.7	12.9	25.8	26.9	26.4	14.1	15.3	13.9
		BEIR VI, EAD	8.7	9.6	9.1	18.9	19.7	19.1	9.9	10.8	9.8
Netherlands											
([18], 2001)	23	Two-mutation carcinogenesis model	-	-	-	-	-	-	2	6	4
Sweden											
([18], 2001)	110	Two-mutation carcinogenesis model	-	-	-	-	-	-	17	24	20
Canada											
([15], 2005)	28	BEIR VI, EAC	-	-	7.3	-	-	13.5	-	-	7.8
([1], 2012)	42	EPA model	15.3	14.3	14.8	29.5	27.8	28.4	16	16	16
([17], 2013)	43	BEIR VI, EAC	-	-	12.3	-	-	21.9	-	-	13.6
France											
([16], 2006)	89	BEIR VI, EAC	-	-	11	-	-	50	-	-	13
		BEIR VI, EAD	-	-	8	-	-	36	-	-	9
		European pooling study	-	-	-	-	-	-	-	-	5
Germany											
([14], 2008)	49	European pooling study	5.0	5.2	-	5.2	5.2	-	-	-	5.0
Switzerland											
([14], 2008)	78	European pooling study	8.2	8.6	-	8.8	8.8	-	-	-	8.3
United Kingdom											
([20], 2009)	21	BEIR VI, EAC	-	-	-	-	-	-	-	-	6.0
		European pooling study	-	-	-	-	-	-	-	-	3.3
Portugal											
([21], 2012)	81	BEIR VI, EAC	25	23	-	40	38	-	27	34	-
		BEIR VI, EAD	18	17	-	31	29	-	20	27	-
South Korea											
([19], 2015)	62	BEIR VI, EAC	18.6	18.5	-	33.2	32.8	-	19.5	28.2	-
		BEIR VI, EAD	-	-	-	-	-	-	13.5	20.4	-
		European pooling study	-	-	-	-	-	-	8.3	8.3	-

The values not presented in papers were left blank

*EAC* exposure-age-concentration model, *EAD* exposure-age-duration model, *EPA* Environmental Protection Agency

**Table 2:** Number of radon-attributable lung cancer deaths per year according to smoking status and gender

Country (reference)	Mean indoor radon (Bq/m^3^)	Model used in risk estimation	Ever-smokers			Never-smokers			Ever- and never-smokers		
			Male	Female	Total	Male	Female	Total	Male	Female	Total
United States											
([2], 1999)	46	BEIR VI, EAC	11300	7600	18900	1200	1700	2900	12500	9300	21800
		BEIR VI, EAD	7900	5400	13300	900	1200	2100	8800	6600	15400
Netherlands											
([18], 2001)	23	Two-mutation carcinogenesis model	-	-	-	-	-	-	90	60	150
Sweden											
([18], 2001)	110	Two-mutation carcinogenesis model	-	-	-	-	-	-	242	178	420
Canada											
([15], 2005)	28	BEIR VI, EAC	-	-	-	-	-	-	-	-	1400
([1], 2012)	42	EPA model	1639	1198	2837	166	258	424	1805	1456	3261
([17], 2013)	43	BEIR VI, EAC	-	-	708	-	-	139	-	-	847
France											
([16], 2006)	89	BEIR VI, EAC	-	-	2578	-	-	759	-	-	3337
		BEIR VI, EAD	-	-	1819	-	-	541	-	-	2361
		European pooling study	-	-	-	-	-	-	-	-	1234
Germany											
([14], 2008)	49	European pooling study	1390	347	1737	32	127	159	1422	474	1896
Switzerland											
([14], 2008)	78	European pooling study	164	54	218	5	8	13	169	62	231
United Kingdom											
([20], 2009)	21	BEIR VI, EAC	-	-	-	-	-	-	1156	888	2044
		European pooling study	-	-	-	-	-	-	637	473	1100
Portugal											
([21], 2012)	81	BEIR VI, EAC	1627	308	1935	143	60	203	1769	369	2138
		BEIR VI, EAD	1183	226	1409	111	46	157	1294	271	1565
South Korea											
([19], 2015)	62	BEIR VI, EAC	-	-	-	-	-	-	26782	13695	40477
		BEIR VI, EAD	-	-	-	-	-	-	18614	9947	28561
		European pooling study	-	-	-	-	-	-	11906	4271	16177

The values not presented in papers were left blank

*EAC* exposure-age-concentration model, *EAD* exposure-age-duration model, *EPA* Environmental Protection Agency

Averaged indoor radon concentrations range from 21 to 110 Bq/m^3^ (arithmetic means), showing considerable variations among countries. Although directly comparing results from different epidemiological studies would be difficult because of methodological differences, countries with high indoor radon concentrations tend to have high estimates of percent AR for lung cancer deaths. The highest estimate of percent AR (20 %) was reported in a Swedish study using two-mutation carcinogenesis model for a country-wide average radon concentration of 110 Bq/m^3^ [18]. The lowest indoor radon concentration (21 Bq/m^3^) was measured in the UK, which also showed the lowest percent AR (3.3 %) of lung cancer deaths given in the model from the European pooling study [20].

Estimates of AR are dependent on the risk model used. Several studies have applied the models proposed by the sixth Biological Effects of Ionizing Radiation (BEIR-VI) Committee [2] for calculating lung cancer deaths attributable to indoor radon exposure in the general population. The BEIR-VI models were developed by reanalyzing the initial combined analysis of 11 cohorts of miners in 1994 [22]. The committee derived two linear excess risk models representing the multiplicative increment in the excess lung cancer risk beyond background levels of radon [2, 23]. Both models take into account time since exposure, the attained age, and either the duration of the exposure (exposure-age-duration, EAD model) or the level of concentration (exposure-age-concentration model, EAC model). Regardless of the chosen model, the estimated numbers are similar, however the EAC model tends to produce higher values than the EAD model [21]. For the US [2] and France [16], overall percent ARs were estimated higher in the EAC model than in the EAD model (13.9 % vs. 9.8 % in the USA and 13 % vs. 9 % in France, respectively). Accordingly, the numbers of lung cancer deaths due to indoor radon were higher when applying the EAC model than the EAD model: in the US, 21,800 lung cancer deaths were probably caused by indoor radon annually in the EAC model and 15,400 in the EAD model, with 3337 in the EAC model and 2361 in the EAD model in France. Although estimates for overall percent AR were not presented in research articles from Portugal [21] and South Korea [19], a similar trend was observed in the overall numbers of lung cancer deaths, which were calculated as a summation of deaths for both genders (Table 2).

A model developed by the European pooling study [8, 9] has also been commonly used to estimate the lung cancer risk due to indoor radon. Unlike the BEIR-VI models, which are subject to uncertainties because of indirect methods extrapolating evidence from miners to the general population [24], the European pooling study utilized data on lung cancer and residential radon from 13 general European population case-control studies (7,148 cases of lung cancers and 14,208 controls) [8], consequently estimating directly lung cancer risk due to indoor radon exposure. The risk derived from the European pooling study was adjusted for age, gender, region of residence, and smoking status. The model of the European pooling study tends to produce lower ARs than the BEIR-VI models. In France, a 5 % (1234 lung cancer deaths) AR was calculated using the model of the European pooling study, regardless of gender and smoking status, which is lower than estimates calculated by the BEIR-VI models [16]. The Advisory Group on Ionising Radiation also reported similar trends in the UK, with percent ARs of 3.0 % using the European pooling study model and 6.0 % using the BEIR-VI model [20]. In South Korea, both males and females showed lower estimates of AR using the European pooling study model than with using the BEIR-VI models [19]. These discrepancies may be due to overestimation of the relevant exposure in older age groups and due to the lack of correction for uncertainties in radon distribution in the BEIR-VI models [14].

Leenhouts and Brugmans [18] applied a two-mutation carcinogenesis model for calculating ARs in the Netherlands and Sweden. This model has previously been used in a number of animal experiments and in a study by Leenhouts [25] to examine the induction of lung cancer by smoking and radon exposure. The model assumes that two mutations play a role in the transformation of a normal somatic stem cell to a malignant cell and the effects of radon exposure and smoking are caused by changes in mutation rates [26]. Chen et al. [1] used a model proposed by the US Environmental Protection Agency (EPA model). This model was devised as a single model from two BEIR-VI models (EAC and EAD models) by assigning risk values midway between the two models [23], since both are equally preferred and it is difficult to choose only one in practice [2]. The two-mutation carcinogenesis model and the EPA model have not been used as commonly as the BEIR-VI models and that from the European pooling study. Furthermore, the two-mutation carcinogenesis model and EPA model were used respectively in each study, and thus, it is impossible to directly compare their AR estimates with those from the other models.

### Attributable risk according to smoking status and gender

Active smoking became the most common cause of lung cancer during the 20th century [27], and several studies have been established that approximately 90 % of all lung cancers occur among smokers [27–31]. Thus, considering the possible interaction with smoking is important when investigating the effect of radon exposure on lung cancer risk.

In general, never-smokers are affected more by radon exposure than smokers, but the absolute risk is higher for smokers than for never-smokers, because of the higher lung cancer rate among smokers [2, 23, 32, 33]. As for the AR of lung cancer deaths due to indoor radon, attributable percentages were higher for never-smokers than ever-smokers; however, greater number of lung cancer deaths due to radon exposure occurred in ever-smokers than in never-smokers (Tables 1 and 2). For example, in the US, the percent ARs of lung cancer deaths due to indoor radon exposure for both genders ranged from 19.1 to 26.4 % among never-smokers and 9.1–12.9 % among ever-smokers, whereas the number of lung cancer deaths reached 2100–2900 for never-smokers and 13,300–18,900 for ever-smokers [2]. Studies in Canada [1, 17] and France showed similar results. Even after analyzing separately by gender, this trend remained consistent in several studies from various countries [1, 2, 14, 21].

In several epidemiological studies, percent ARs have been estimated to be slightly higher for females than for males regardless of smoking status (Table 1). However, the differences in the attributable percentages between genders were similar after stratifying populations according to smoking status. These results may be due to the lower proportion of female smokers compared to male smokers in most countries. The estimated numbers of lung cancer deaths attributable to radon exposure among both ever- and never-smokers were higher for males than for females (Table 2). This is likely due to the high smoking rates in males, and thus, the overall number of lung cancer deaths was higher in males than in females. Only for never-smokers, namely under the condition of excluding influence by smoking, more radon-induced lung cancer deaths occurred among females than among males in most studies.

### Effects of indoor radon mitigation

The measured indoor radon levels follow a lognormal distribution in general. In other words, most individuals are exposed to low concentrations of radon in their homes. Evidence from studies on general populations suggests that chronic exposure to radon at low doses can cause lung cancer [8, 34]. Therefore, it is needed to reduce the indoor radon concentrations to lower level to prevent more lung cancers due to radon exposure.

Table 3 shows the estimated percentages and numbers of lung cancer deaths attributable to radon that could be prevented if all homes above given radon concentrations were effectively remediated. The effects of radon mitigation on lung cancer were assessed in studies conducted in the US [2], Germany [14], and Canada [1, 17]. In the US, under the EAC model, mitigating radon levels in homes at or above 148 Bq/m^3^, the EPA action level [13], would result in an estimated reduction in lung cancer mortality of 4.2 % if indoor radon were completely eliminated, 3.7 % if homes were mitigated to 0–148 Bq/m^3^, or 1.7 % if homes were remediated to exactly 148 Bq/m^3^ [2]. In Germany, reducing radon levels below 100 Bq/m^3^ (WHO guideline [4]) in homes would prevent 302 lung cancer deaths (15.9 % of all lung cancer deaths attributable to radon) every year [14]. At mitigation levels of 200 and 400 Bq/m^3^ (European action level for new and old houses), 143 (7.5 %) and 68 (3.6 %) deaths could be potentially avoided, respectively. In Canada, out of total 3261 radon-induced lung cancer deaths nationwide, 1704 (52.3 %) can be prevented per year if all homes with radon above 100 Bq/m^3^ were remediated to outdoor levels, and 927 (28.4 %) at the Canadian action level of 200 Bq/m^3^ [1]. Additionally, it was predicted that reducing indoor radon levels to outdoor level for all homes above 100 and 200 Bq/m^3^ would prevent 233 (28 %) and 91 (11 %) radon-attributable lung cancer deaths, respectively, in Ontario, Canada [17].

**Table 3:** Preventable lung cancer deaths if all homes above mitigation level of radon concentration were remediated

Country (reference)	Estimation model	Background levels (Bq/m^3^)	Mitigation level of indoor radon concentration				
			Radon-attributable lung cancer deaths (n, %) that can be prevented				
United States			37 Bq/m^3^	74 Bq/m^3^	148 Bq/m^3^		
([2], 1999)	BEIR-VI, EAC	0	11.0 %	7.8 %	4.2 %		
		< Mitigation level	9.2 %	6.5 %	3.7 %		
		Mitigation level	6.8 %	4.0 %	1.7 %		
	BEIR-VI, EAD	0	7.7 %	5.5 %	3.1 %		
		< Mitigation level	6.5 %	4.7 %	2.7 %		
		Mitigation level	4.9 %	2.8 %	1.2 %		
Germany			100 Bq/m^3^	150 Bq/m^3^	200 Bq/m^3^	250 Bq/m^3^	400 Bq/m^3^
([14], 2008)	European pooling study	9 (outdoor level)	302, 15.9 %	197, 10.4 %	143, 7.5 %	115, 6.1 %	68, 3.6 %
Canada			100 Bq/m^3^	200 Bq/m^3^	400 Bq/m^3^	600 Bq/m^3^	800 Bq/m^3^
([1], 2012)	EPA model	outdoor level	1704, 52.3 %	927, 28.4 %	345, 10.6 %	165, 5.1 %	90, 2.8 %
Ontario, Canada			50 Bq/m^3^	100 Bq/m^3^	150 Bq/m^3^	200 Bq/m^3^	
([17], 2013)	BEIR-VI, EAC	10–30	389, 46 %	233, 28 %	149, 18 %	91, 11 %	

*EAC* exposure-age-concentration model, *EAD* exposure-age-duration model, *EPA* Environmental Protection Agency

The results from several studies suggest that setting mitigation levels of indoor radon lower can prevent more lung cancer deaths. Therefore, strategies for radon remediation are needed to reduce the risk of radon-related lung cancer. Such strategies should consider technological difficulties, success rates, and costs, because radon sources and radon transport mechanisms may have a considerable influence on the cost-effectiveness [4].

### Uncertainty

There are many sources of uncertainty in estimating AR. These uncertainties stem mainly from the weakness of the model used and the various factors influencing indoor radon concentrations.

The BERI-VI models have several sources of uncertainty [2]. Among these sources, extrapolation of results from the studies of miners to assess the risk of lung cancer for general populations is a critical issue of uncertainty. Using exposure-response relations determined among underground miners to assess the risk in the general population underlines some of the differences between these two populations [16]. Miners are generally exposed to higher levels of radon than the general population. Sex and age distributions are also different between both populations: miners are almost all men of working age, whereas the general population comprises men and women of all ages. Exposure and risk can also be modified by various physical and biological factors such as ventilator flow, breathing frequency, tracheobronchial configuration, and an individual’s physical size [21]. Moreover, smoking-related risks in miners have been reported to be different from those in the general population, and many miners are exposed to various carcinogens other than radon, such as arsenic [4]. Despite these uncertainties, most studies have had to assume that the lung cancer risk due to exposure to indoor radon is close to that observed among miners, because of a lack of appropriate data.

The two-mutation carcinogenesis model also has some weaknesses [18]. This model is a simplification of the development of lung cancer caused by smoking and radon exposure, but the real process is more complex. Furthermore, there are a large number of parameters that have to be determined when fitting the model to data, and parameters are assumed to be dependent on exposure to external agents, but not on age. These imply statistical uncertainties in the parameters, which are difficult to quantify, because of the interplay between the parameters and model assumptions.

The estimation of indoor radon concentrations is also associated with numerous uncertainties. Indoor radon concentrations depend on various factors, such as the soil, building materials, house type, and ventilation. Therefore, concentrations can vary between houses and even from room to room in the same house due to some conditions such as ventilation practices [8, 35]. Indoor radon concentrations also vary substantially between and within regions [36]. Seasons are related to variations in radon concentrations within homes, with the highest levels in winter and the lowest in summer. As well, annual average radon concentrations are subject to substantial random year-to-year variations related to numerous factors such as weather patterns and occupant behaviors [4]. The uncertainties introduced by these factors need to be addressed adequately using statistical corrections.

Additionally, methodological issues for measurements are sources of uncertainty in estimating indoor radon concentrations. Potential radon exposure misclassification can arise from detector’s measurement errors and localization choices within a home, inaccessible data on previously occupied homes, failure to link radon concentrations with subject mobility, and measuring radon gas concentration as a surrogate for radon progeny exposure [37]. Unfortunately, the impact of these uncertainties on AR estimations is very difficult to quantify. However, if the misclassification were to be non-differential between cases and controls, the observed results tend to be underestimated.

## Conclusions

Radon is the great public health threat conveyed by indoor air. Epidemiological studies have confirmed that radon in homes increases the risk of lung cancer in the general population. Among the carcinogens of lung cancer, radon is the second leading cause after smoking. Of all lung cancer deaths, from 3 to 20 % are attributable to indoor radon exposure worldwide, depending on the average radon concentration in the country and on the method of calculation. Radon is much more likely to cause lung cancer in ever-smokers than in never-smokers, but it is the primary cause of lung cancer among never-smokers. A large portion of radon-induced lung cancer deaths are caused by radon concentrations below commonly used reference levels, because the majority of general population are exposed to low and moderate level of indoor radon. These observations imply that effective measures to prevent and reduce indoor radon concentrations should be developed and included in national radon control programs.
